# 3D imaging of sex-sorted bovine spermatozoon locomotion, head spin and flagellum beating

**DOI:** 10.1038/s41598-018-34040-3

**Published:** 2018-10-23

**Authors:** Mustafa Ugur Daloglu, Francis Lin, Bryan Chong, Daniel Chien, Muhammed Veli, Wei Luo, Aydogan Ozcan

**Affiliations:** 1 Electrical and Computer Engineering Department, University of California, Los Angeles, CA 90095 USA; 2 Bioengineering Department, University of California, Los Angeles, CA 90095 USA; 3 California NanoSystems Institute (CNSI), University of California, Los Angeles, CA 90095 USA; 4 Department of Surgery, David Geffen School of Medicine, University of California, Los Angeles, CA 90095 USA

## Abstract

With the advent of sperm sex sorting methods and computer-aided sperm analysis platforms, comparative 2D motility studies showed that there is no significant difference in the swimming speeds of X-sorted and Y-sorted sperm cells, clarifying earlier misconceptions. However, other differences in their swimming dynamics might have been undetectable as conventional optical microscopes are limited in revealing the complete 3D motion of free-swimming sperm cells, due to poor depth resolution and the trade-off between field-of-view and spatial resolution. Using a dual-view on-chip holographic microscope, we acquired the full 3D locomotion of 235X-sorted and 289 Y-sorted bovine sperms, precisely revealing their 3D translational head motion and the angular velocity of their head spin as well as the 3D flagellar motion. Our results confirmed that various motility parameters remain similar between X- and Y-sorted sperm populations; however, we found out that there is a statistically significant difference in Y-sorted bovine sperms’ preference for helix-shaped 3D swimming trajectories, also exhibiting an increased linearity compared to X-sorted sperms. Further research on e.g., the differences in the kinematic response of X-sorted and Y-sorted sperm cells to the surrounding chemicals and ions might shed more light on the origins of these results.

## Introduction

Differences in the swimming characteristics of X-chromosome and Y-chromosome bearing sperm cells have been an important research topic for researchers, where earlier studies suggested a difference in their swimming velocities and head volume due to the smaller size of the Y-chromosome compared to the X-chromosome^1–3^. Based on this hypothesis, researchers tried to separate X-bearing and Y-bearing sperms with gradient solutions, assuming that Y-bearing sperm would reach the target zone first because of their higher velocity and a greater ability to penetrate fluid interfaces^4,5^. Although accepted at the time, a proof for the validity of these assumptions were never available^6,7^.

The difference in the genetic content of the X and Y sperm was highlighted with fluorescent labeling and flow cytometry^8^ to be able to separate the two cell populations in rabbit^9^, swine^10^, human^7^, and in livestock including cattle and sheep^11–13^. Sex selection has an especially important economic significance for livestock (e.g., dairy farmers)^14^ where the accuracy of sex separation can reach up to 90%^15,16^ and the process has already been commercialized^17^. With such reliable methods of separating the X and Y sperm becoming available, along with the advent of computer-aided sperm analysis (CASA) systems^18–22^, various accurate comparative studies have been made. Following the implementation of these successful sorting techniques, studies were performed using e.g., optical microscopy techniques^23,24^ and atomic force microscopy^25^. These 2D studies did not reveal any statistically significant differences in the dimensions or dimensional distributions as well as the 2D motion parameters between X and Y sperm^3,23,26^. However a statistically significant difference of 4% in the linearity of the 2D trajectories was reported, X-sorted sperm cells exhibiting more linear trajectories compared to Y-sorted sperm cells^6^. It should be noted that the 2D tracking of sperms does not reveal the complete information about their 3D swimming behavior, especially when the sperm cells are confined in shallow chambers for optical imaging with conventional microscopes. When imaged in deeper chambers (i.e. >100 µm), sperm cells exhibit 3D swimming patterns^27–33^ which could reveal further differences in the swimming properties of X and Y sorted sperms. In addition to the 3D translational head motion of the swimming of sperm cells, other factors such as the 3D rotational motion of the head and the 3D flagellar beating are also critical^33^, which could reveal further differences in the swimming characteristics of X-sorted and Y-sorted sperms^6^. However, such a comparison has been unavailable to researchers due to the limitations of conventional lens-based microscopes. The trade-off between the resolution and field-of-view (FOV) and poor depth-resolution of optical microscopy tools do not allow for 3D tracking of sperm cells in large numbers within deep chambers. As a computational alternative^32^ to conventional optical microscopy, on-chip holographic imaging^34–38^ permits high-throughput 3D tracking of sperm cells with sub-micron 3D positioning accuracy, revealing rotational motion of the sperm head as well as its 3D flagellar beating^28–30,33^.

**Figure 1:**
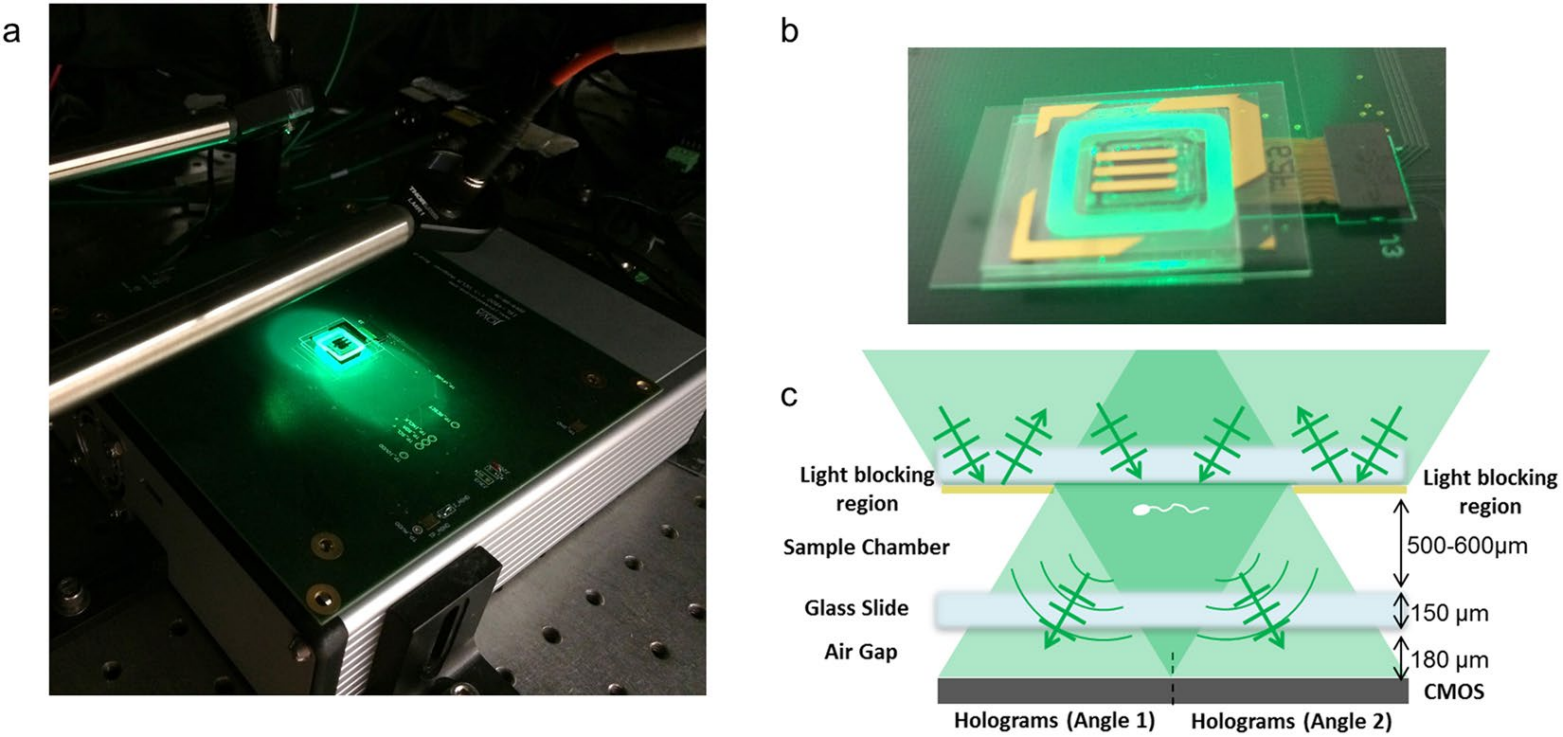
(**a**) Our dual-view on-chip holographic imaging platform consists of a CMOS image sensor connected to a frame grabber operating at ~300 fps and two fiber-coupled LEDs emitting green light (~525 nm center-wavelength), placed in mirror-symmetry with a ~18° tilt^33^. (**b**) The sample chamber placed above the image sensor with a periodic light-blocking structure as the top layer^33^. (**c**) The periodic light-blocking structure spatially separates the pair of holographic projections across the image sensor, fully utilizing the dynamic range of the CMOS imager, achieving better sensitivity to image weakly-scattering flagellar structures of sperm cells^33^.

Here we used a dual-view on-chip holographic microscope^33^ to compare the full 3D swimming properties of 524 sex-sorted bovine sperms (i.e., 235 X-sorted and 289 Y-sorted) over a large depth-of-field of ~500–600 µm. To reveal the 3D translational and rotational head motion as well as the 3D flagellar beating characteristics of sperm locomotion, our set-up consisted of two oblique fiber-coupled light-emitting diodes (LEDs) emitting green light placed in mirror symmetry, a complementary metal–oxide–semiconductor (CMOS) image sensor and a periodic light-blocking structure (Fig. 1). Two holographic projections containing information of the sperm head and flagellum from the perspective of the two LEDs are generated and spatially separated across the sensor chip active area by the periodic light-blocking structure to fully utilize the dynamic range of the sensor chip, which boosts our sensitivity. A sequence of frames is then captured, with the sensor operating at ~300 frames per second^33^, sufficiently high to capture the sperm motion without temporal undersampling^19^. Each acquired holographic pair is then digitally reconstructed generating a pair of 2D projections of the sperm head and flagellum. The 3D sperm head position and the 3D flagellar structure are obtained through tracing and triangulation using the angles of the light sources, and the 3D head orientation is tracked through the successive phase-wrapping events that occur when the thick side of the sperm head is aligned with one of the light sources^33^.

With all these information sources, the 3D swimming patterns and all the relevant sperm motility parameters were calculated and compared, along with the angular velocity of the sperm head spin and the flagellar beating, for the X and Y sorted bovine sperm cells, which has been previously underexplored due to the limitations of conventional lens-based microscopes used in CASA systems, limiting the sperm motility analysis into 2D within shallow imaging chambers constricting the 3D motion of sperm cells. Here we investigate if a comparative 3D motility analysis of free-swimming sex-sorted bovine sperm cells, with sufficiently deep imaging chambers, could reveal previously undetected differences in conventional sperm motility parameters due to the 2D confinement of sperm cells. Furthermore, our dual-view on-chip imaging platform enables us to record and compare additional motility parameters related to the head spin, 3D flagellar beating and the 3D structure of the swimming trajectories, that could only be captured in a 3D imaging configuration, allowing a comparison between X-sorted and Y-sorted sperm populations, in this regard for the first time.

## Results and Discussion

Our dual-view on-chip holographic imaging platform consists of a CMOS image sensor with a 1.12 µm pixel-pitch and two fiber-coupled LEDs operating at ~525 nm center wavelength with a ~20 nm bandwidth, which are placed in mirror-symmetry at ~18° angle (Fig. 1a). A ~500–600 µm thick micro-channel, housing the sample, is placed very close to the sensor active area (<200 µm) with a periodic light-blocking structure at the top (Fig. 1a,b). This structure enables full sensor dynamic-range utilization by spatially separating the pair holographic projections of each sperm within the imaging volume (Fig. 1c), encoding the spatial information of both the sperm head and sperm tail^33^.

In order to remove the floating debris or non-motile sperms, background subtraction is applied to the raw holographic frames as the first step. The holographic projection pairs of the remaining motile objects in each frame are then digitally reconstructed, with respect to the corresponding illumination source, resulting in two different perspectives of the sperm head and flagellum, both with amplitude and phase information (resulting from the holographic reconstruction). A growing chain model is used to trace both 2D flagellum projections in the stronger phase reconstructions, starting from the head-flagellum junction, by constantly searching for maximum signal and placing nodes with 3 µm intervals until the signal falls below the background noise level. After smoothening the flagellum tracings, each node pair from the two projections are triangulated to find the corresponding 3D node position with sub-micron 3D positioning accuracy^28,39^ starting from the head-flagellum junction, using the angle between the two light sources. By processing each frame within a stack recorded at ~300 fps, detailed information of the 3D head position and the 3D flagellar beating pattern is extracted from a large number of sperms using our dual-view on-chip holographic imaging platform.

In addition to revealing the sperm head position and 3D flagellar structure as a function of time, our platform is also capable of tracking the 3D orientation of the sperm head and measuring its angular velocity across the full trajectory, by monitoring the consecutive phase wrapping events on the phase reconstructions of the sperm head. This rotation of the sperm head along its long axis has been relatively underexplored and can be precisely measured using our platform^33,40^. A phase wrapping event occurs when the thicker side of the sperm head is aligned with one of the corresponding light sources, as the large difference in optical path length generates a strong phase signal. The 3D head orientation as well as the angular velocity of the sperm head is determined by tracking these consecutive phase wrapping events in both projections, providing complete 3D information on sperm swimming^33^.

**Figure 2:**
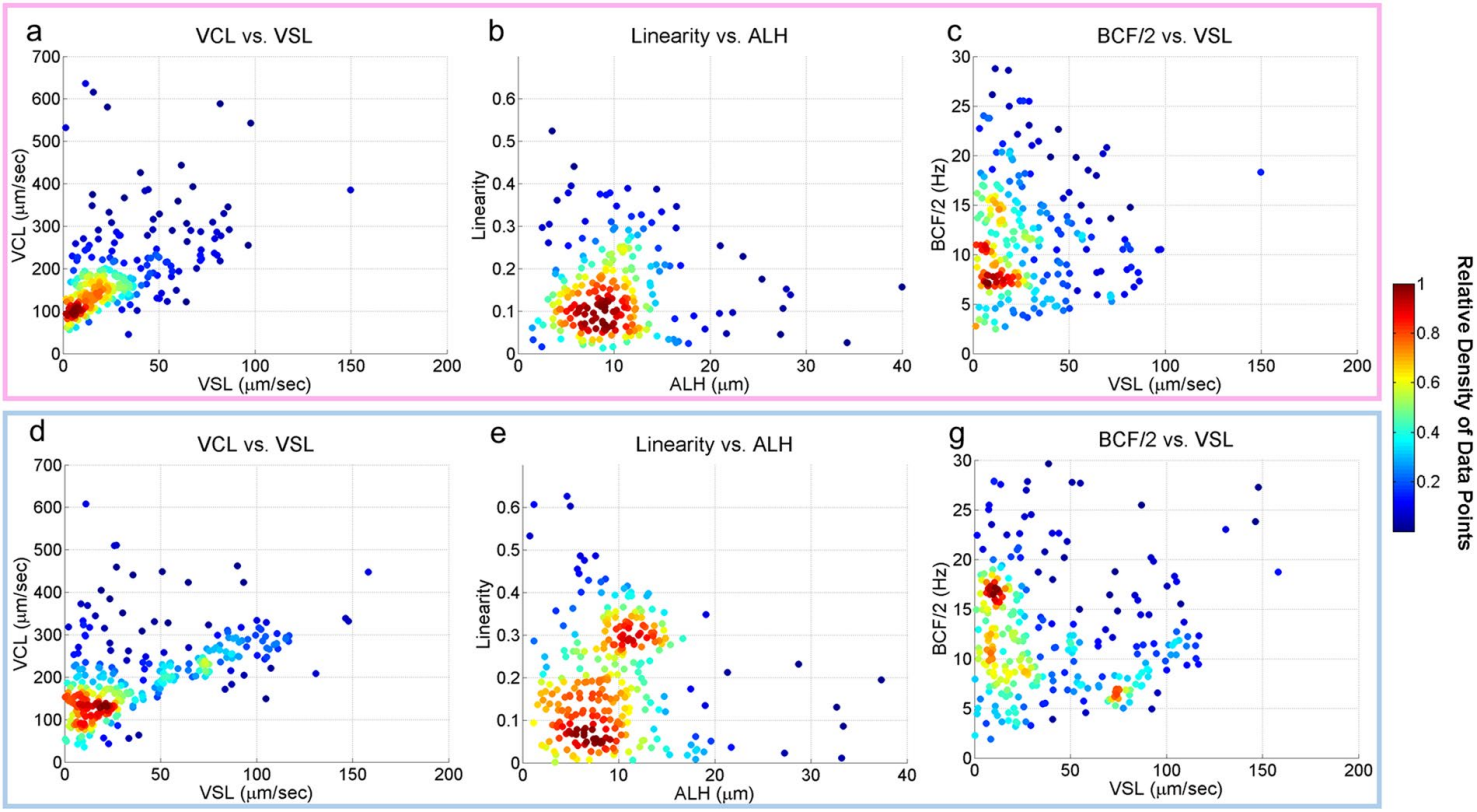
Head motion parameters of 235 X-sorted (enclosed in pink – (**a**–**c**) and 289 Y-sorted (enclosed in blue – (**d**,**e** and **g**) bovine sperms. The color code represents the relative density of the data points. The curvilinear velocity (VCL), straight-line velocity (VSL), beat cross-frequency (BCF) and amplitude of lateral head displacement (ALH) for the X-sorted and Y-sorted bovine sperms show similar values. It was also observed that Y-sorted bovine sperms have more linear trajectories compared to X-sorted bovine sperms (P < 0.05) (**b** and **e**).

**Figure 3:**
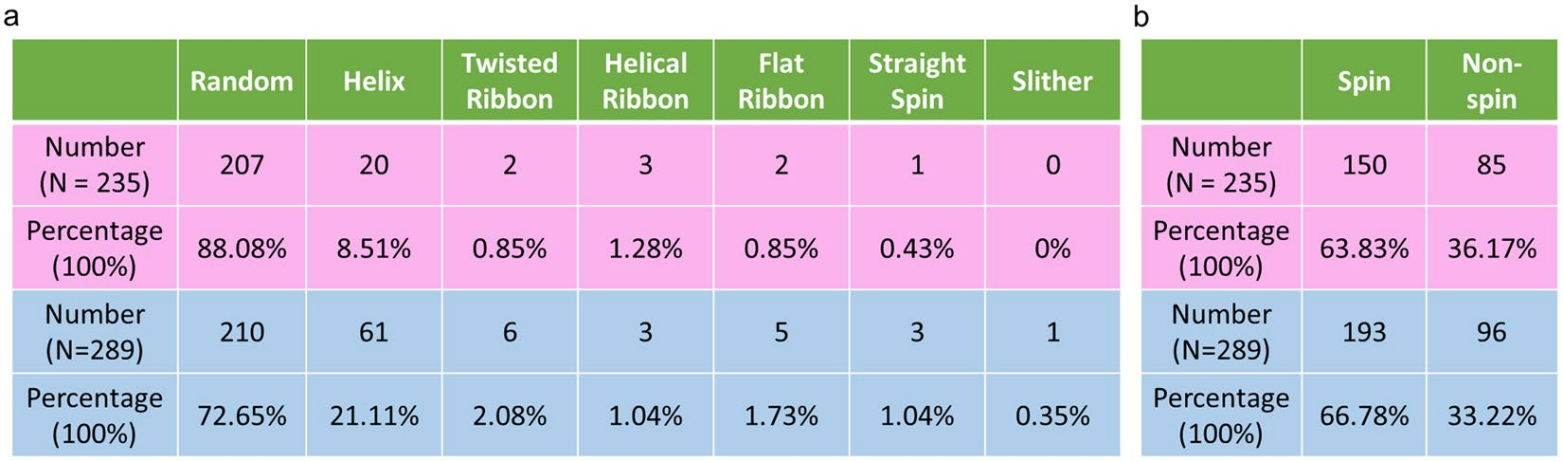
(**a**) Categories for 3D swimming trajectories exhibited by X-chromosome bearing (pink) and Y-chromosome bearing (blue) bovine sperm cells. The preference for helical trajectories was observed to be significantly higher in Y-sorted bovine sperms compared to X-sorted ones. (**b**) The ratio of sperm cells that exhibited head spin during its locomotion was similar for both X-chromosome bearing (pink) and Y-chromosome bearing (blue) bovine sperm cells.

**Figure 4:**
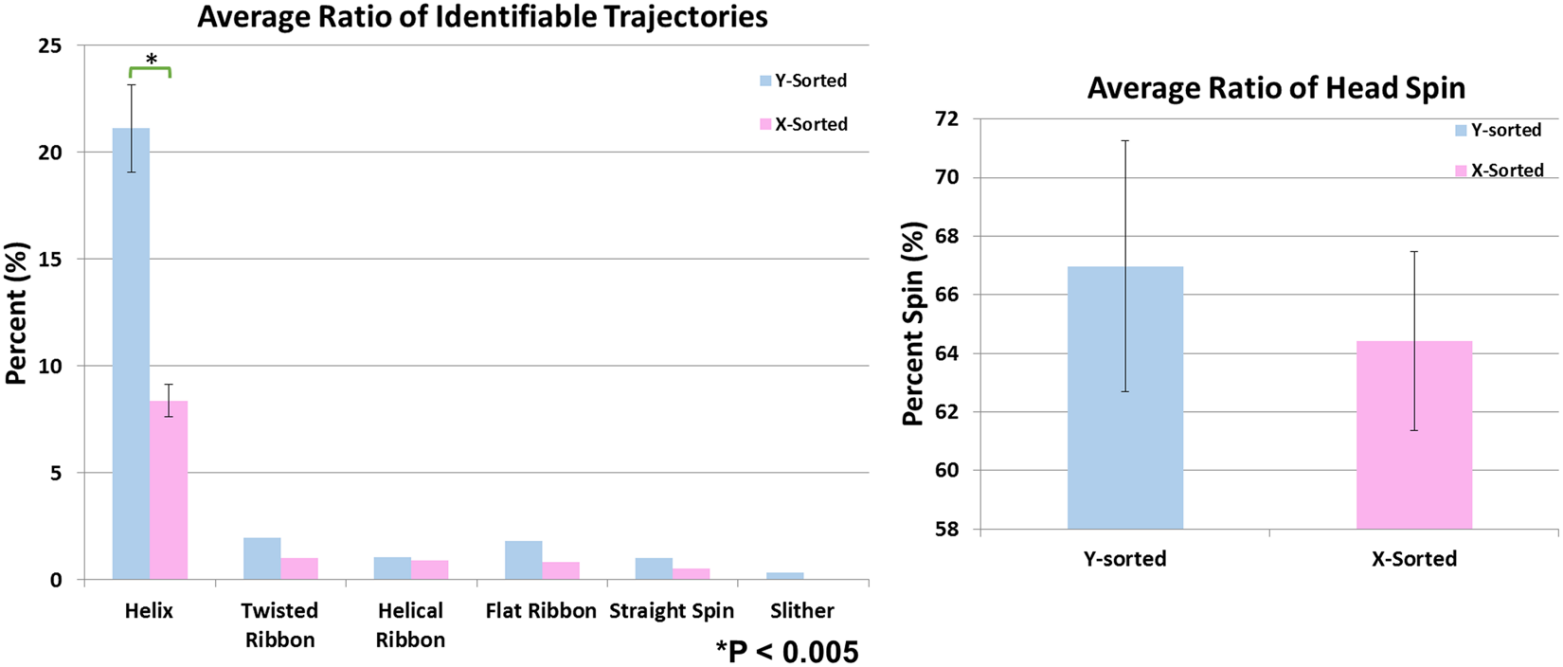
(**a**) The mean ratio of the 3D swimming trajectory categories across three separate experiments for both X-chromosome bearing (pink) and Y-chromosome bearing (blue) bovine sperm cells shows a statistically significant difference in the helix mode swimming category (P < 0.005). (**b**) The mean ratio of head spin across three separate experiments is similar for both sperm populations. The bars represent the standard deviation within the three separate experiments.

**Figure 5:**
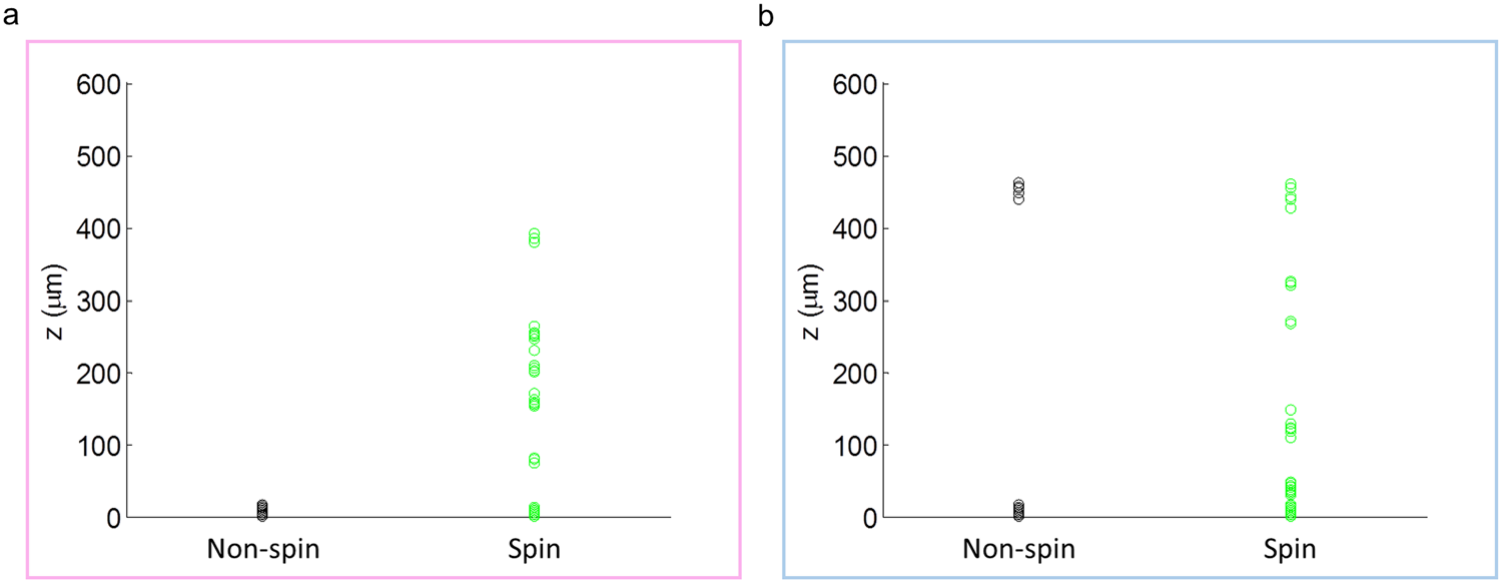
The vertical distribution of the sperm cells across our imaging chamber. (**a**) X-sorted sperm cells and (**b**) Y-sorted sperm cells. The sperm cells that did not exhibit head spin (black dots) are very close to the top or bottom chamber surfaces. The cells that did exhibit head spin (green dots) showed a more continuous height distribution within the channel.

**Figure 6:**
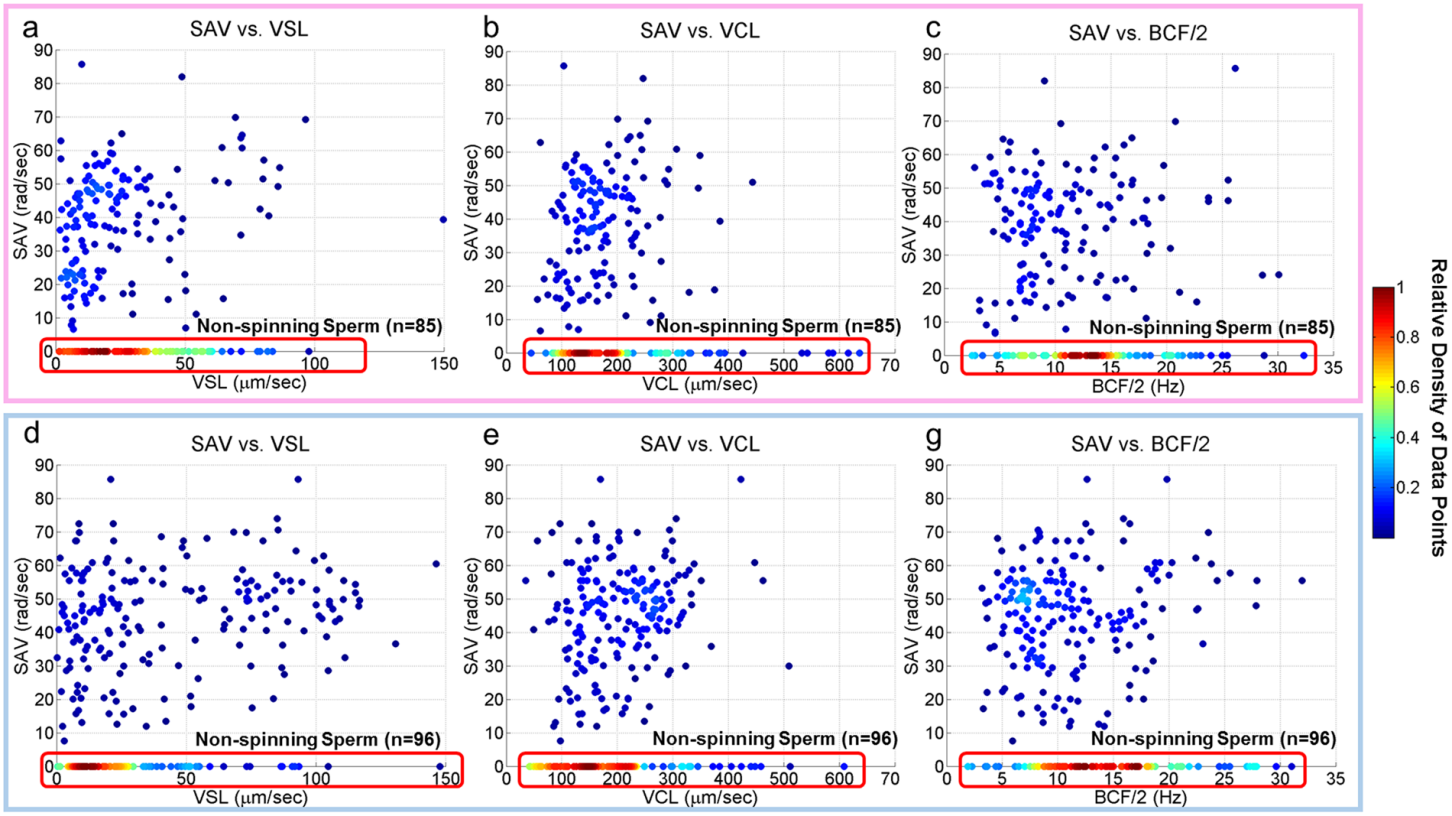
(**a**) Spin angular velocity (rad s^−1^) and head motion parameters of 235 X-sorted (enclosed in pink – (**a**–**c**) and 289 Y-sorted (enclosed in blue – (**d**,**e** and **g**) bovine sperms. The color code represents the relative density of the data points. The spin angular velocity (SAV) of the two bovine sperm populations showed no statistically significant difference.

**Figure 7:**
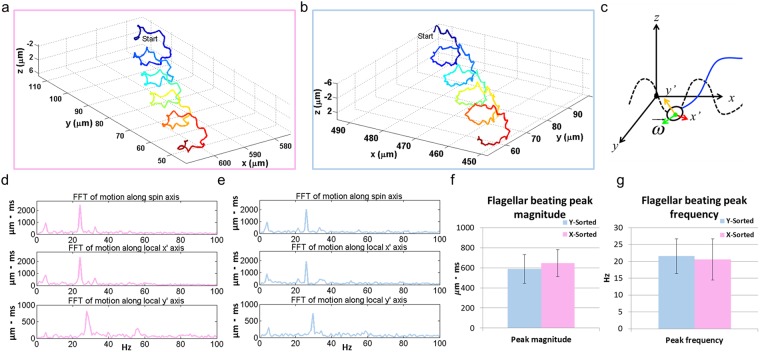
(**a**,**b**) A helical sperm trajectory from an X-sorted (enclosed in pink) and Y-sorted (enclosed in blue) sperm, respectively. (**c**) The local coordinate system used to analyze the true 3D kinematic of the flagellar beating from the perspective of an observer sitting on and traveling with the sperm head. The thicker side of the sperm head corresponds to the ***x’***-axis, the thinner side corresponds to the ***y’***-axis and the $\vec{{\boldsymbol{\omega }}}$-axis extends from the head-flagellum junction towards the tip of the head. (**d**,**e**) The frequency domain analysis of the motion of a flagellar node ~40 µm away from the head-flagellum junction (in arc-length) for the helical trajectories shown in (**a**,**b**) respectively. The dominant beating frequency was observed to be ~24 Hz for the X-chromosome bearing sperm and ~26 Hz for the Y-chromosome bearing sperm with similar amplitudes. (**f**,**g**) The peak beating amplitude and frequency was observed to be similar for both sperm populations.

Utilizing this unique computational imaging platform, we captured and reconstructed 235 X-sorted and 289 Y-sorted bovine sperm trajectories with the information of the 3D head translational and rotational motions as well as the 3D flagellar beating patterns (see Supplementary Movies S1 and S2), providing detailed information for a comparative study of sex-sorted sperm. Conventional sperm motility parameters, including the curvilinear velocity (VCL), straight-line velocity (VSL), linearity, amplitude of lateral head displacement (ALH) and beat cross-frequency (BCF)^19,41^ were calculated in 3D and compared (Fig. 2) along with different categories of 3D swimming trajectories (Figs 3, 4 and 5) as well as the spin angular velocity (SAV) for X and Y sperms (Fig. 6). In these comparisons, the observation frequency of helical trajectories by Y-chromosome bearing bovine sperms was found to be significantly more than the X-chromosome sperms (Fig. 4), for which a flagellar beating comparison was also made (Fig. 7).

In agreement with the previous findings^6^, the translational head motion parameters (Fig. 2) showed that there were no significant differences in the swimming velocities of the X-sorted and Y-sorted bovine sperm cells (Fig. 2a,d). The ALH and BCF also showed similar results for both sexes (Fig. 2b,c,e and g); however the Y-sorted sperms showed a higher linearity compared to X-sorted sperms (P < 0.05) (Fig. 2b,e), which is in contrast to previous studies which suggested that X-sorted sperm exhibit more linear trajectories^6^. These earlier reports, however, calculated these parameters for 2D sperm trajectories in shallow observation chambers, which do not take into account the 3D nature of sperm locomotion. Compared to other species, sex-sorted bovine sperm is observed to exhibit the highest average VCL (~197 μm/sec) and average ALH (~10.2 μm), where the VCL and ALH are lower for sex-sorted semen from boar (~122 μm/sec and ~4.2 μm)^42^, ram (~150 μm/sec and ~6.9 μm)^43^ and stallion (~69 μm/sec and ~3.0 μm)^44^, while the average VSL (~36 μm/sec) is closer to stallion (~27 μm/sec)^44^ and lower compared to boar (~73 μm/sec)^42^ and ram (~75 μm/sec)^43^. The average BCF (~12 Hz) of sex-sorted bovine sperm also appears to be closer to the average BCF of stallion (~7 Hz)^44^, lower compared to ram (~32 Hz)^43^.

In addition to these translational head motion parameters reported in Fig. 2, 3D trajectories of sex-sorted bovine sperm cells have been categorized and compared according to their swimming mode^28–30,33^ which include: random (88.08% for X-sperm and 72.65% for Y-sperm), helix (8.51% for X-sperm and 21.11% for Y-sperm), twisted ribbon (0.85% for X-sperm and 2.08% for Y-sperm), helical ribbon (1.28% for X-sperm and 1.04% for Y-sperm), flat ribbon (0.85% for X-sperm and 1.73% for Y-sperm), straight spin (0.43% for X-sperm and 1.04% for Y-sperm) and slithering mode (0% for X-sperm and 0.35% for Y-sperm) (Fig. 3a). A similar comparison is provided in Fig. 3b for head spin: 63.83% for X-sperm and 66.78% for Y-sperm.

The major difference between the random category and the others is that the random trajectories show more arbitrary shapes where the other categories exhibit more periodic shapes^28,29,33^. A lower average linearity (~0.17) is observed in random trajectories compared to the average linearity of helical trajectories (~0.21) where both the average VSL and VCL appear to be slightly larger for helical trajectories (~44 μm/sec and ~217 μm/sec) compared to random trajectories (~33 μm/sec and ~193 μm/sec), which is also reflected in their average SAV (~38 rad/sec and ~26 rad/sec respectively). Among the trajectories that are observed quite rarely, twisted ribbon and helical ribbon show similar values to helical trajectories where the flat ribbon category shows lower average VSL and linearity (~14 μm/sec and ~0.11) and the straight spin shows higher average VSL and linearity (~53 μm/sec and ~0.29), although the average ALH appears to be similar for all categories (~10–11 μm). The sperm cells that exhibit slithering trajectories exhibit highly linear trajectories (~0.6), but do not show head spin (SAV = 0) as they swim very close to the chamber surface^33^.

Among all the 3D swimming categories, a statistically significant difference (P < 0.005) was *only* observed in the helix mode 3D swimming category (see Fig. 4a). This could be one of the reasons for the higher linearity observed in Y-chromosome bearing bovine sperm compared to X-chromosome bearing bovine sperm (Fig. 2), since helical trajectories exhibit higher linearity^28,33^. While the biophysical reasons for these observed differences are not exactly known, further studies which incorporate the kinematic responses of X-sorted and Y-sorted sperm cells to the surrounding medium^45–52^ (e.g., calcium ions^53–55^ present in the buffer that we used^56^) and the differences in molecular features of X and Y sperm populations^57^ would be critical to better understand the origins of these observed differences.

Besides the helical mode of 3D swimming category, the head spin percentages of the X-sorted and Y-sorted bovine sperm also did not show a significant difference (Fig. 4b). An interesting observation is that the sperms that did not exhibit head spin were very close the bottom or top surface of our observation chamber (Fig. 5), which once again highlights the importance of deeper channels to observe 3D swimming patterns of sperms. The spin angular velocity (SAV) of the two bovine sperm populations were also compared (Fig. 6), showing no statistically significant difference. Although the SAV is not conventionally used in sperm motility analysis at the moment since current CASA systems rely on 2D sperm tracking, it could have future significance in assessing sperm quality for clinical and research purposes with the potential emergence and use of 3D CASA systems.

Next, we investigated the 3D spatio-temporal kinematics of the flagellar beating observed in the helical trajectories of X-sperms and Y-sperms (Fig. 7a,b), within a local coordinate system (Fig. 7c) defined with respect to the sperm head (i.e. from a perspective of an observer seated on the sperm head looking towards the flagellum), which was made possible with our dual-view imaging platform by tracking the head orientation throughout the sperm locomotion^33^. The 3D motion of the flagellum node was analyzed in the frequency domain (Fig. 7d,e), showing the dominant beating frequency and its magnitude (Fig. 7f,g) which was compared for both sperm populations. No statistically significant difference was observed in these flagellar beating parameters extracted from X-sorted and Y-sorted sperms as detailed in Fig. 7.

Our results show that while many parameters are quite similar between the two populations, there is a statistically significant difference in the observation frequency of helical trajectories for Y-chromosome bearing sperms, along with an increased linearity compared to X-bearing ones. The reasons behind these observations are not known, and further research on the differences in chemical and biological pathways governing sperm locomotion, which depend on various molecular mechanisms^57,58^, could provide new clues, benefiting from some of the unique measurement capabilities of our dual-view holographic sperm imaging platform.

## Methods

### Sample Preparation

Sex-sorted bovine sperm was obtained frozen (between −190 °C and −200 °C) in ~0.25 µL straws (STgenetics, Navasota TX), which was thawed in warm water (37 °C) for 30 seconds and gently placed on the 500 µL top layer (40% BoviPure –60% BoviDilute, Nidacon, Sweden) of a gradient solution with a 500 µL bottom layer (80% BoviPure –20% BoviDilute)^56^ within a centrifuge tube (Falcon, 25 mL Fisher Scientific). After 15 minutes centrifugation (Fisher Scientific) at 300 g, the pellet at the bottom containing the motile sperm cells is gently extracted and re-suspended within a 1 mL of BoviWash (Nidacon, Sweden), which is then centrifuged for an additional 5 minutes under 300 g. The pellet at the bottom of the tube is then gently extracted (~10 µL) and re-suspended in 90 µL BoviWash, serving as the base for further dilutions (100–200X) suitable for holographic imaging, and placed in imaging chambers constructed from regular glass cover slips (150 µm thick, Fisher Scientific) for the bottom surface and with the periodic light-blocking structure for the top surface, with a ~500–600 µm thick silicone spacer (3 M Company) sandwiched in between, which defines the micro-channel height for sperm locomotion. The periodic light-blocking structures were constructed by depositing ~4 mm long, ~450 µm wide reflective light-blocking stripes with a periodicity of ~900 µm^33^. All the samples were prepared and stored within an incubator set to 37 °C, and all the materials used, including the sperm wash media, were preheated prior to the experiments.

### Dual-view Holographic On-chip Imaging Platform

Our dual-view on-chip holographic imaging platform consists of a de-capped CMOS image sensor (IMX135, Sony Corporation, Tokyo, Japan) with a pixel-pitch of 1.12 µm operating at ~300 fps, through a custom designed frame grabber, which transfers the frames to a computer (Dell T3600) via a high-speed PCIe interface. Two fiber-coupled LEDs emitting green light (~525 nm center wavelength with a ~20 nm bandwidth) are placed in mirror symmetry with an angle of incidence of ~18° (Fig. 1a). The sample chamber is placed very close (~180 µm) to the sensor, with a periodic light-blocking structure at the top to spatially separate the pair of holographic projections obtained for each sperm cell across the sensor (Fig. 1b), fully utilizing the dynamic range and preventing crosstalk between the hologram pairs (Fig. 1c). Operating at ~300 fps, the platform captures the complete 3D motion of the bovine sperm cells, including both the head motion and rotation along with the 3D structure of the flagellum without temporal under sampling^33^.

### Holographic Reconstruction and Tracking

Background subtraction (using the moving average of ~100–200 frames as background) is applied to remove immotile sperm cells and other debris from the raw frames, isolating the hologram pairs corresponding to the motile sperms^33^. Each holographic projection is then digitally back-propagated to the correct height with the corresponding angle of incidence and wavelength using the angular spectrum approach^59^. A 2D tail tracing, composed of equally spaced nodes with 3 µm intervals, is iteratively fitted to each of the phase reconstructions starting from the head-flagellum junction, searching for the maximum signal over a uniform angular range of ±40° at each step. The fitting process is terminated once the signal levels fall below the noise threshold, and the 2D tail tracings are further smoothened to a node length of ~0.19 µm^33^. The 3D structure of the flagellum is then obtained by pairing and triangulating nodes along both projections that share the same arc-length from the head-flagellum junction, resulting in a physically accurate 3D reconstruction of the sperm flagellum^33^. The 3D orientation of the sperm head is also determined throughout the sperm locomotion by tracking the phase-wrapping events that occur sequentially in both phase reconstruction pairs, as the thicker side of the sperm head gets aligned with each of the corresponding light sources. Tracing these successive phase wrapping events, the angular velocity as well as the spatial orientation of the sperm head is determined, which was used to define a local coordinate system from a perspective of an observer seated on the sperm head (***x***′-axis along the thicker side, ***y***′-axis along the thinner side and $\vec{{\boldsymbol{\omega }}}$-axis extending from the head-flagellum junction towards the tip of the head)^33^. This local perspective was essential in the investigation of the true 3D spatio-temporal kinematics of the flagellum, reported in Fig. 7. It takes ~5.1 seconds to process each frame using a single CPU core, resulting in approximately 86 minutes for processing a complete trajectory composed of 300 frames; however a 10–20 fold speed up would be expected with a GPU implementation of our reconstruction algorithms^33^. The statistical significance of the differences in linearity and the preference for helical trajectories were tested using a t-test^60^, with three separate experiments for each sex, where the minimum number of sperm cells per experiment was 59.

## Conclusions

Using a dual-view on-chip imaging platform operating at ~300 fps, we tracked and compared the complete 3D swimming motion of 235 X-sorted and 289 Y-sorted bovine sperm cells, which includes the translational and rotational motion of the sperm head and the 3D flagellar beating observed from a perspective local to the sperm head, revealing the true spatio-kinematics of the sperm locomotion. A comparison of the conventional sperm motility parameters of X and Y sperms showed no statistically significant difference in the velocity parameters (VCL and VSL), in agreement with previous findings that the both sperm populations have similar swimming speeds. Enabled by our dual-view on-chip imaging platform, the head spin velocities and the 3D trajectory categories were also analyzed and compared to each other. Although both sperm populations exhibited similar rotational speeds, a statistically significant difference was observed in the percentage of helical trajectories, suggesting that Y-chromosome bearing bovine sperm cells have a higher preference for this category, along with a higher linearity compared to X-chromosome bearing cells. A further investigation of these helical mode sperm cells revealed no difference in 3D flagellar beating behavior of these sperms. Although the exact reason for the difference in the preference for helical trajectories is unclear and this difference is not suitable for sperm sorting purposes, further studies which incorporate the differences in the kinematic response of X-sorted and Y-sorted sperm cells to the surrounding chemicals and ions could be critical for understanding the origins of these observations. We believe our dual-view holographic imaging platform offers unique opportunities for such micro-swimmer tracking applications.

## Electronic supplementary material

Supplementary Movie 1

Supplementary Movie 2

## References

[1] Shettles LB (1960). Nuclear Morphology of Human Spermatozoa. Nature.

[2] Shettles LB (1961). After Office Hours Conception and Birth Sex Ratios: A Review. Obstet. Gynecol..

[3] Grant VJ (2006). Entrenched misinformation about X and Y sperm. BMJ.

[4] Ericsson RJ, Langevin CN, Nishino M (1973). Isolation of Fractions rich in Human Y Sperm. Nature.

[5] Beernink FJ, Dmowski WP, Ericsson RJ (1993). Sex preselection through albumin separation of sperm. Fertil. Steril..

[6] Penfold LM (1998). Comparative motility of X and Y chromosome-bearing bovine sperm separated on the basis of DNA content by flow sorting. Mol. Reprod. Dev..

[7] Johnson LA (1993). Preimplantation diagnosis: Gender preselection in humans? Flow cytometric separation of X and Y spermatozoa for the prevention of X-linked diseases. Hum. Reprod..

[8] Hamano K (2007). Sex Preselection in Bovine by Separation of X- and Y-Chromosome Bearing Spermatozoa. J. Reprod. Dev..

[9] Johnson LA, Flook JP, Hawk HW (1989). Sex Preselection in Rabbits: Live Births from X and Y Sperm Separated by DNA and Cell Sorting. Biol. Reprod..

[10] Johnson LA (1991). Sex Preselection in Swine: Altered Sex Ratios in Offspring following Surgical Insemination of Flow Sorted X- and Y-Bearing Sperm. Reprod. Domest. Anim..

[11] Johnson L (2000). Sexing mammalian sperm for production of offspring: the state-of-the-art. Anim. Reprod. Sci..

[12] de Graaf SP, Beilby KH, Underwood SL, Evans G, Maxwell WMC (2009). Sperm sexing in sheep and cattle: The exception and the rule. Theriogenology.

[13] Seidel GE (1999). Insemination of heifers with sexed sperm. Theriogenology.

[14] Seidel GE (2003). Economics of selecting for sex: the most important genetic trait. Theriogenology.

[15] Wheeler MB (2006). Application of sexed semen technology to *in vitro* embryo production in cattle. Theriogenology.

[16] Grant VJ, Chamley LW (2007). Sex-Sorted Sperm and Fertility: An Alternative View. Biol. Reprod..

[17] Garner DL, Seidel GE (2008). History of commercializing sexed semen for cattle. Theriogenology.

[18] Mortimer ST (1997). A critical review of the physiological importance and analysis of sperm movement in mammals. Hum. Reprod. Update.

[19] Mortimer ST (2000). CASA—Practical Aspects. J. Androl..

[20] Liu, J., Leung, C., Lu, Z. & Sun, Y. Human Sperm Tracking, Analysis, and Manipulation. In *Smart Materials-Based Actuators at the Micro/Nano-Scale* (Springer New York, 2013).

[21] Amann RP, Waberski D (2014). Computer-assisted sperm analysis (CASA): Capabilities and potential developments. Theriogenology.

[22] Mortimer ST, van der Horst G, Mortimer D (2015). & others. The future of computer-aided sperm analysis. Asian J. Androl..

[23] Zavaczki Z (2006). Dimensional assessment of X-bearing and Y-bearing haploid and disomic human sperm with the use of fluorescence *in situ* hybridization and objective morphometry. Fertil. Steril..

[24] Hossain, A. M., Barik, S. & Kulkarni, P. M. Lack of Significant Morphological Differences Between Human X and Y Spermatozoa and Their Precursor Cells (Spermatids) Exposed to Different Prehybridization Treatments. *J. Androl*. 5 (2001). 11191075

[25] Carvalho JO, Silva LP, Sartori R, Dode MAN (2013). Nanoscale Differences in the Shape and Size of X and Y Chromosome-Bearing Bovine Sperm Heads Assessed by Atomic Force Microscopy. PLoS ONE.

[26] Ferrara M (2015). Label-Free Imaging and Biochemical Characterization of Bovine Sperm Cells. Biosensors.

[27] Corkidi G, Taboada B, Wood CD, Guerrero A, Darszon A (2008). Tracking sperm in three-dimensions. Biochem. Biophys. Res. Commun..

[28] Su T-W, Xue L, Ozcan A (2012). High-throughput lensfree 3D tracking of human sperms reveals rare statistics of helical trajectories. Proc. Natl. Acad. Sci..

[29] Su, T.-W. *et al*. Sperm Trajectories Form Chiral Ribbons. *Sci. Rep*. **3** (2013). 10.1038/srep01664 PMC3630328 23588811

[30] Su T-W, Choi I, Feng J, Huang K, Ozcan A (2016). High-throughput analysis of horse sperms’ 3D swimming patterns using computational on-chip imaging. Anim. Reprod. Sci..

[31] Jikeli JF (2015). Sperm navigation along helical paths in 3D chemoattractant landscapes. Nat. Commun..

[32] Daloglu MU, Ozcan A (2017). Computational imaging of sperm locomotion. Biol. Reprod..

[33] Daloglu MU (2018). Label-free 3D computational imaging of spermatozoon locomotion, head spin and flagellum beating over a large volume. Light Sci. Appl..

[34] Mudanyali O (2010). Compact, light-weight and cost-effective microscope based on lensless incoherent holography for telemedicine applications. Lab. Chip.

[35] Gorocs Z, Ozcan A (2013). On-Chip Biomedical Imaging. IEEE Rev. Biomed. Eng..

[36] Greenbaum A (2012). Imaging without lenses: achievements and remaining challenges of wide-field on-chip microscopy. Nat. Methods.

[37] McLeod E, Ozcan A (2016). Unconventional methods of imaging: computational microscopy and compact implementations. Rep. Prog. Phys..

[38] Ozcan A, McLeod E (2016). Lensless Imaging and Sensing. Annu. Rev. Biomed. Eng..

[39] Su T-W (2010). Multi-angle lensless digital holography for depth resolved imaging on a chip. Opt. Express.

[40] Babcock DF, Wandernoth PM, Wennemuth G (2014). Episodic rolling and transient attachments create diversity in sperm swimming behavior. BMC Biol..

[41] Di Caprio G (2014). 4D tracking of clinical seminal samples for quantitative characterization of motility parameters. Biomed. Opt. Express.

[42] García EM (2007). Improving the fertilizing ability of sex sorted boar spermatozoa. Theriogenology.

[43] de Graaf SP (2007). The influence of antioxidant, cholesterol and seminal plasma on the *in vitro* quality of sorted and non-sorted ram spermatozoa. Theriogenology.

[44] Balao da Silva CM (2013). Sex sorting increases the permeability of the membrane of stallion spermatozoa. Anim. Reprod. Sci..

[45] Morisawa M (1994). Cell signaling mechanisms for sperm motility. Zoolog. Sci..

[46] Gil PI (2008). Chemotactic response of frozen-thawed bovine spermatozoa towards follicular fluid. Anim. Reprod. Sci..

[47] Ho H-C, Suarez SS (2001). Hyperactivation of mammalian spermatozoa: function and regulation. Reproduction.

[48] Kaupp UB, Kashikar ND, Weyand I (2008). Mechanisms of Sperm Chemotaxis. Annu. Rev. Physiol..

[49] Chang H, Suarez SS (2010). Rethinking the Relationship Between Hyperactivation and Chemotaxis in Mammalian Sperm1. Biol. Reprod..

[50] Spehr M (2004). Particulate Adenylate Cyclase Plays a Key Role in Human Sperm Olfactory Receptor-mediated Chemotaxis. J. Biol. Chem..

[51] Eisenbach M, Giojalas LC (2006). Sperm guidance in mammals — an unpaved road to the egg. Nat. Rev. Mol. Cell Biol..

[52] Kaupp UB (2003). The signal flow and motor response controling chemotaxis of sea urchin sperm. Nat. Cell Biol..

[53] Böhmer M (2005). Ca2+ spikes in the flagellum control chemotactic behavior of sperm. EMBO J..

[54] Suarez SS (2008). Control of hyperactivation in sperm. Hum. Reprod. Update.

[55] Ho H-C, Granish KA, Suarez SS (2002). Hyperactivated Motility of Bull Sperm Is Triggered at the Axoneme by Ca2+ and Not cAMP. Dev. Biol..

[56] Samardzija M (2006). A comparison of BoviPure® and Percoll® on bull sperm separation protocols for IVF. Anim. Reprod. Sci..

[57] De Canio M (2014). Differential protein profile in sexed bovine semen: shotgun proteomics investigation. Mol BioSyst.

[58] Chen X (2012). Identification of differentially expressed proteins between bull X and Y spermatozoa. J. Proteomics.

[59] Goodman, J. W. *Introduction to Fourier optics*. (Roberts & Co, 2005).

[60] Fay, D. S. A biologist’s guide to statistical thinking and analysis. *WormBook* 1–54 10.1895/wormbook.1.159.1 (2013). 10.1895/wormbook.1.159.1 PMC3880567 23908055

